# Nigella sativa Improves Glycemic Control and Ameliorates Oxidative Stress in Patients with Type 2 Diabetes Mellitus: Placebo Controlled Participant Blinded Clinical Trial

**DOI:** 10.1371/journal.pone.0113486

**Published:** 2015-02-23

**Authors:** Huda Kaatabi, Abdullah Omar Bamosa, Ahmed Badar, Abdulmohsen Al-Elq, Bodour Abou-Hozaifa, Fatma Lebda, Akram Al-Khadra, Sameeh Al-Almaie

**Affiliations:** 1 Department of Physiology, College of Medicine, University of Dammam, Dammam, Saudi Arabia; 2 Department of Medicine, College of Medicine, University of Dammam, Dammam, Saudi Arabia; 3 Department of Family Medicine, College of Medicine, University of Dammam, Dammam, Saudi Arabia; Pennington Biomedical Research Center, UNITED STATES

## Abstract

**Background and Objective:**

Oxidative stress plays an important role in pathogenesis of diabetes mellitus and its complications. Our previous study has shown glucose lowering effect produced by 3 months supplementation of Nigella sativa (NS) in combination with oral hypoglycemic drugs among type 2 diabetics. This study explored the long term glucose lowering effect (over one year) of NS in patients with type 2 diabetes mellitus on oral hypoglycemic drugs and to study its effect on redox status of such patients.

**Methods:**

114 type 2 diabetic patients on standard oral hypoglycemic drugs were assigned into 2 groups by convenience. The control group (n = 57) received activated charcoal as placebo and NS group (n = 57) received 2g NS, daily, for one year in addition to their standard medications. Fasting blood glucose (FBG), glycosylated hemoglobin (HbA_1c_), C- peptide, total antioxidant capacity (TAC), superoxide dismutase (SOD), catalase (CAT), glutathione and thiobarbituric acid reactive substances (TBARS) at the baseline, and every 3 months thereafter were determined. Insulin resistance and β-cell activity were calculated using HOMA 2 calculator.

**Results:**

Comparison between the two groups showed a significant drop in FBG (from 180±5.75 to 180±5.59 in control Vs from 195±6.57 to 172 ±5.83 in NS group), HbA1c (from 8.2±0.12 to 8.5±0.14 in control VS from 8.6±0.13 to 8.2±0.14 in NS group), and TBARS (from 48.3±6.89 to 52.9 ±5.82 in control VS from 54.1±4.64 to 41.9 ±3.16 in NS group), in addition to a significant elevation in TAC, SOD and glutathione in NS patients compared to controls. In NS group, insulin resistance was significantly lower, while β-cell activity was significantly higher than the baseline values during the whole treatment period.

**Conclusion:**

Long term supplementation with Nigella sativa improves glucose homeostasis and enhances antioxidant defense system in type 2 diabetic patients treated with oral hypoglycemic drugs.

**Trial Registration:**

Clinical Trials Registry-India (CTRI) CTRI/2013/06/003781

## Introduction

Diabetes mellitus (DM) is a common chronic disease that affects people of all ages and races and imposes a large economic burden on the health care system. DM is characterized by chronic elevation of blood glucose which is a central factor in the production of reactive oxygen species (ROS) that, in turn, promote cellular damage and contribute to the development and progression of diabetic complications.[1, 2]

Pancreatic β-cells are particularly susceptible to damage by oxidative stress when exposed to chronic hyperglycemia [3] because they are low in free-radical quenching enzymes.[4] Further, ROS can suppress the insulin response and contribute to the development of insulin resistance, a key pathological feature of type 2 DM.[5] Therefore, adequate glycemic control is essential for preventing complications associated with type 2 diabetes. Intensive glucose-lowering strategy has been found to reduce the risk of microvascular endpoints in patients with type 2 diabetes by up to 25%.[6] Moreover, clinical trials showed that a 1% decrease in HbA_1c_ reduces cardiovascular complications by 14%.[7]

Nigella sativa (NS) seeds, an annual Ranunculaceae herbaceous plant, have been used in folk medicine to treat diabetes.[8] The glucose lowering and antidiabetic effects of NS has been reported in different diabetic animal models.[9, 10] Benhaddou-Andaloussi et al. [11] have demonstrated that NS ethanol extract exhibits a remarkable ability in vitro to concomitantly increase insulin secretion, induce proliferation of pancreatic β-cells, and stimulate glucose uptake in skeletal muscle and fat cells. In another study, the same investigators reported that treatment with NS ethanol extract, in diabetic ‘*Meriones shawi*’ rats, caused a progressive normalization of glycaemia, albeit slower than that of metformin controls and exerted an insulin-sensitizing action, compared to diabetic controls. [12]

The protective effect of NS has been attributed to its strong antioxidant properties, which are related to its ability to scavenge various reactive oxygen species, including superoxide radical anion and hydroxyl free radicals,[13] to block lipid peroxidation and to enhance antioxidant enzymes.[14,15]

In spite of the extensive discussion on the hypoglycemic and antioxidant effects of NS in diabetic animal models,[15,16] there is still scant evidence about these effects in humans and especially in diabetics. Najmi *et al* [17] reported that NS oil (2.5 mL twice daily for 6 weeks) has a beneficial effect on fasting blood glucose, total cholesterol, and LDL-cholesterol in obese diabetics, suggesting that NS oil is effective as an ‘add-on’ therapy in patients of insulin resistance syndrome. The results of a more recent study carried out in our department indicated that Nigella sativa in a dose of 2 g/day for 12 weeks might be a beneficial adjuvant to oral hypoglycemic agents in type 2 diabetic patients.[18] However, there is still a concern about persistence of these effects upon long term use of NS. Therefore, the objective of this study was to investigate the effect of NS supplementation, for one year, on glycemic control and oxidant—antioxidant status in type 2 diabetic patients treated with oral hypoglycemic drugs.

## Materials and Methods

### Setting, approval and registration

This study was carried out at Department of Physiology, College of Medicine, University of Dammam, Kingdom of Saudi Arabia from May 2009 to January 2012. The study was approved by the Research and Ethical committee of the University of Dammam. This department has an established research line on Nigella sativa with more than a dozen papers published. The dosages used in this study were established in a predecessor study carried out in 2004–2007.[18] At that time registration of clinical trials was not mandatory. Later on our group had an understanding that Nigella sativa is classified as a herb or functional food and therefore it does not come under the scope of mandatory registration of clinical trials. In the meanwhile the patient recruitment had already commenced. As soon as this confusion was clarified especially after publication of CONSORT extension for herbal medicinal interventions we swiftly proceeded with registration of all of our ongoing trials. We confirm that now all our ongoing trials related to Nigella sativa are registered. This particular trial was registered as a clinical trial in the clinical trial registry of India (No. CTRI/2013/06/003781) available at (http://www.ctri.nic.in/Clinicaltrials/pmaindet2.php?trialid=6054).

### Patient selection and consent

Patients were recruited from outpatient diabetes clinic of the University Hospital by convenience sampling. The inclusion criteria were patients with uncontrolled type 2 diabetes (based on two successive readings three months apart of HbA_1c_of >7%), age between 18 and 60 years, using standard oral hypoglycemic drugs regularly (glibenclamide, metformin, rosiglitazone) and consenting for intervention and follow up. While those with HbA_1c_ > 9%, insulin therapy, body mass index (BMI)>40, major cardiovascular problems (i.e., coronary artery disease, valvular diseases, heart failure), uncontrolled hypertension, renal failure, hepatic failure, and those who had compliance less than 90%, or changed their standard medications during the one year study were excluded.

A written informed consent was taken from the volunteers. The study design was explained to the prospective subjects specifically mentioning the additional medication or placebo to be taken, their responsibilities including regular blood sugar testing, regular follow up dates, calibration of glucometer blood collection at follow up visits etc.

### Drugs

Nigella sativa L. seeds as powder in capsules of 500 mg (Bioextract. Private Limited,Sri Lanka) were used in the study in a dose of 2 g/day. Dose selection was based on a study by Bamosa et al [18], where it was found to be effective in reducing blood sugar.

Activated charcoal capsules (260 mg) similar in size and color to the capsules of NS (Arkopharma Pharmaceutical Laboratories Carros, France) were used as placebo.

### Study design

A total of 114 patients with type 2 DM (63 males and 51 females), who fulfilled the above inclusion and exclusion criteria, were assigned by random allocation (using a list of numbers generated by table of random numbers) into two groups.

Control group (n = 57, 30 males) received placebo and NS group (n = 57, 33 males) received NS. The drugs were given to the patients in two divided doses, daily, for one year. The participants did not know about the content of capsules (placebo or NS).

In addition, the patients of both groups continued to use their own oral hypoglycemic drugs, as adjusted by their doctors, in addition to N. sativa or placebo (activated charcoal). Out of 114 patients 98 were on sulfonylureas and metformin, 16 on metformin alone.

**Fig 1:**
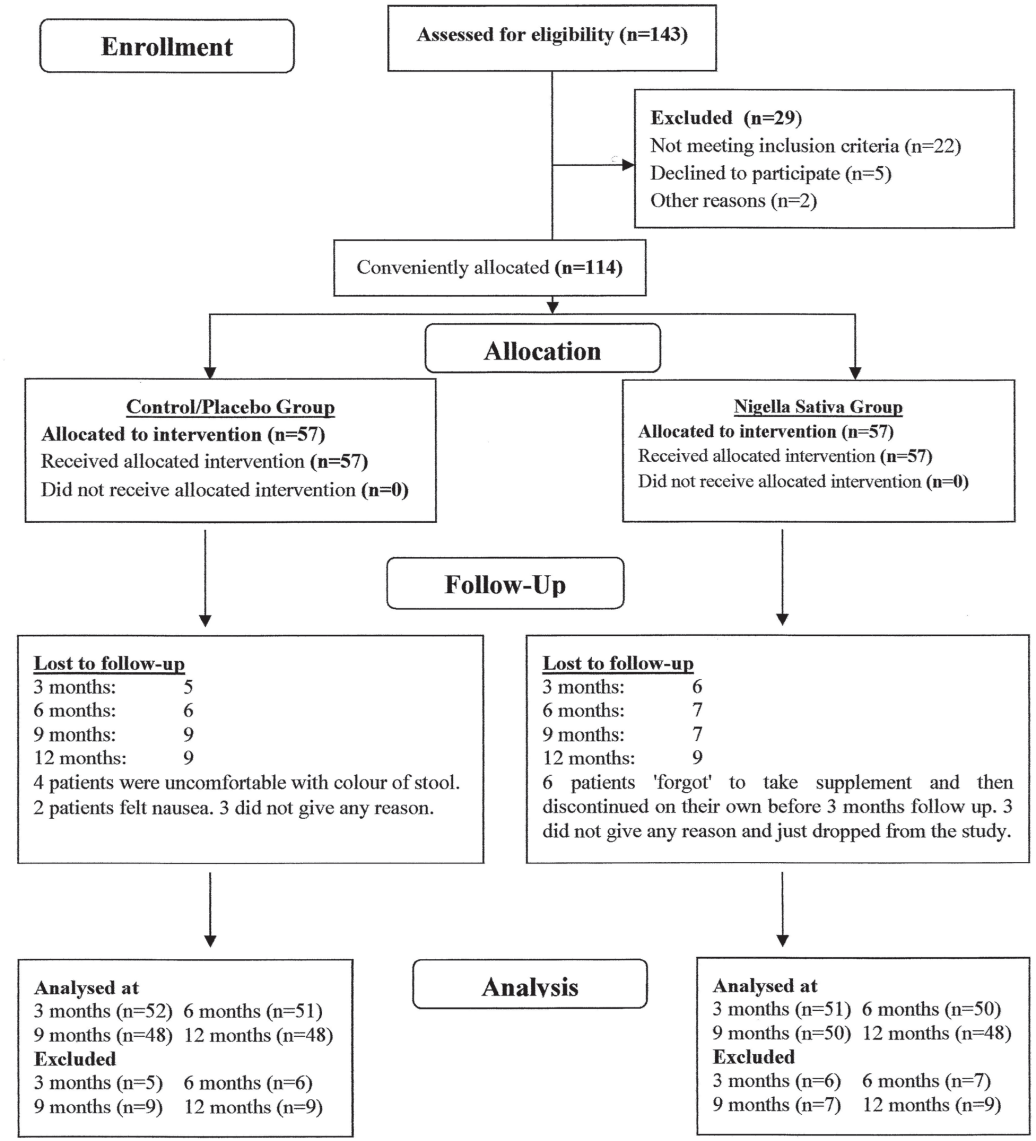
Flow of Patients from enrollment to the end of study.

The flow of patients in study from recruitment to endpoint is shown in Fig. 1.

Four visits each 3 months apart (3, 6, 9 and 12 months) after starting the treatment were scheduled for follow up and the patients were contacted by phone in between the scheduled visits to check for any changes in their medications, or any untoward symptoms.

Prior to treatment, detailed history of the patients was taken and physical examination was carried out.

Data including age, gender, body mass index (BMI) and duration of diabetes were recorded. Glycemic variables including fasting blood glucose (FBG), glycosylated hemoglobin (HbA_1c_), C- peptide, the antioxidant enzymes; superoxide dismutase (SOD), catalase (CAT), together with total Antioxidant capacity (TAC), and levels of glutathione, and lipid peroxidation markers, thiobarbituric acid reactive substances (TBARS) were measured before intervention (baseline) and every 3 months thereafter till the end of the study period.

Insulin resistance (IR) and β-cell function were calculated using computer software HOMA2 (Homeostasis Model Assessment calculator, released by Oxford University UK in 2004) utilizing the FBS and C- Peptide levels, that is based on the formulas of Levy et al[19], (where IR is normal if ˂2.5, marker of IR if ≥2.5).

The patients were supplied with self-monitoring glucometers of the same make (Accu—Chek Go, Roche Diagnostic GmbH, Germany). Calibration of glucometers against standards was carried out before handing over the equipment and then at each follow up visit. After orientation with the recording method, the patients were asked to check their blood sugar once a week, first after 8 hours fasting and then 2 hours after meal (post prandial). The mean of all the weekly readings for a month was considered as the monthly average.

### Blood sample collection

Blood was collected after 12 hours period of fasting by venepuncture, between 08.00 and 09.00 am. Patients were asked not to smoke or engage in physical activity for 30 minutes prior to blood sampling. The first sample of blood (4ml) was collected into plain tubes and the separated serum was used to determine glucose and C-peptide. Another blood sample (4ml) was collected into Ethylene-Diamine-Tetraacetic-Acid (EDTA)-coated tubes and the hemolysates were then used for estimation of HbA_1c_. A third blood sample (4ml) was collected into plain tube, allowed to clot for at least half an hour and then centrifuged at 2000 rpm for 15 minutes at—4°C. The top serum was pipetted into small aliquots and stored at—80°C to be used for antioxidant assays later.

### Analytical methods

Serum fasting blood glucose was assayed on autoanalyzer (Dimension Clinical Chemistry System, Dimension Max. Germany), using Flex reagent cartridges, supplied by Dade Behring, Germany. HbA_1c_ was assayed on autoanalyzer “Hb Gold Analyzer”, using Gold Reagent Kit- HbA_1c_, provided by Drew Scientific Ltd. Germany. Serum C- peptide was assayed on autoanalyzer by Immulite C-peptide Kit, provided by EURO/DPC Ltd. UK. The antioxidant enzymes and TBARS were assayed on ELx 800 automated microplate reader (BIO-TEK Instrument Inc. USA), using ELISA kits supplied by Cayman Chemical Company, USA.

### Statistical analysis

Statistical analyses were performed using the Statistical Package for Social Sciences (SPSS) version 19. As there were multiple measurements (3 months apart) for each parameter as well as missing values, therefore a linear mixed model for analysis was constructed for analysis. The data points used for analysis were Pre intervention (baseline), 3 months, 6 months, 9 months and 12 months after intervention. Data were transformed from wide format (one case per row) to long format (one observation per case per row). In the linear mixed model statistics ‘descriptive statistics’ were selected for summary statistics and ‘parameter estimates’ for model statistics. After identifying the dependent variables we specified fixed (age, gender, BMI and duration of disease) and random (individual subject) factors and covariates to create a mixed model. The relationship between repeated measures was defined as a random effect for each dependent variable and repeated covariance was marked as ‘unstructured’ initially. Later on several correlation structures including ‘compound symmetry’, first order autoregressive and Toeplitz were assessed for each model. We used Akaike’s Information criteria (AIC) as measure of model fit. The model with the smallest AIC was considered the best fit. Data were presented as mean ± standard error of the mean (SEM). Significant results were marked. P value was considered significant if P ≤0.05.

## Results

The baseline values of age, duration of disease, BMI, FBG, HbA_1c_, TAC, SOD, CAT, glutathione and TBARS for all patients included in the study are presented in table 1. No significant differences were found between the control and NS groups in the baseline values for all parameters. The BMI did not vary significantly in either of the groups during the one year course of study.

**Table 1:** Comparison of the baseline data between the control (placebo) group and Nigella sativa group.

Parameter (Mean ± SEM)	Control group (n = 57)	N. sativa group (n = 57)	P value
**Age (years)**	46.12 ± 0.85	46.82 ± 1.14	0.62
**Duration of disease (years)**	5.96 ± 0.57	7.25 ± 0.51	0.09
**Body mass index**(**kg/m** ^**2**^)	31.83 ± 0.52	30.48 ± 0.53	0.07
**Fasting blood glucose (mg/dL)**	180.89 ± 5.44	195.95 ± 6.28	0.07
**Hemoglobin A**_**1c**_ **(%)**	8.23 ± 0.12	8.56 ± 0.12	0.06
**Total antioxidant capacity (mM)**	2.44 ± 0.14	2.16 ± 0.15	0.17
**Superoxide dismutase activity (U/ml)**	2.26 ± 0.22	1.78 ± 0.19	0.09
**Catalase activity (nmol/min/ml)**	68.17 ± 4.72	56.22 ± 4.10	0.06
**Glutathione (μM)**	3.22 ± 0.21	3.64 ± 0.24	0.19
**TBARS (μM)**	47.77 ± 6.40	52.50 ± 4.21	0.54

n = number of patients TBARS: thiobarbituric acid reactive substances.

**Fig 2:**
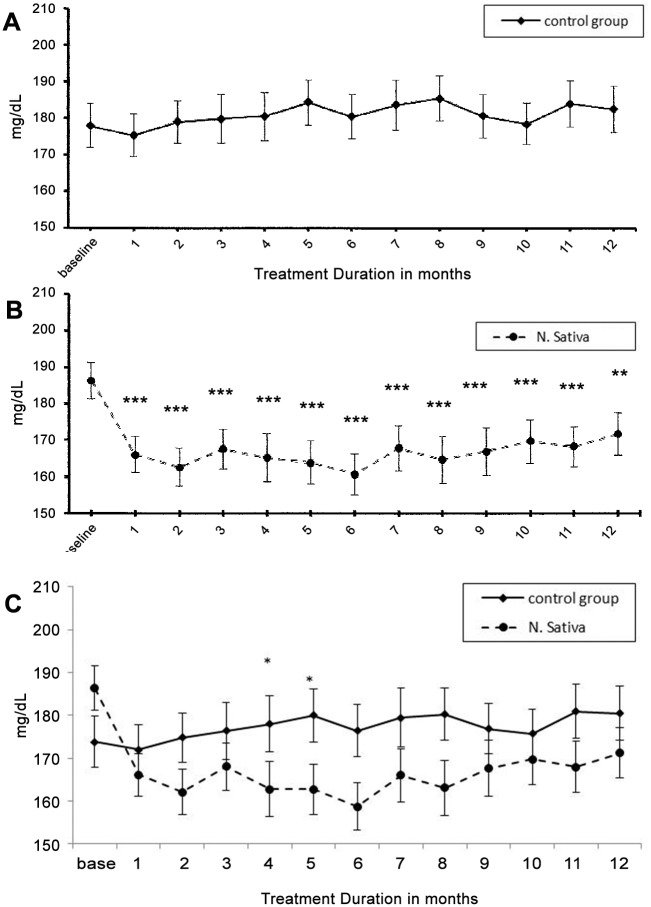
Changes in Fasting blood glucose (Mean±SEM) measured by self- monitoring glucometers, in the control (placebo) group and N. sativa group, compared to the corresponding baseline values using linear mixed model. Difference is significant at * P < 0.05, ** P <0.01 and *** P < 0.001.

**Fig 3:**
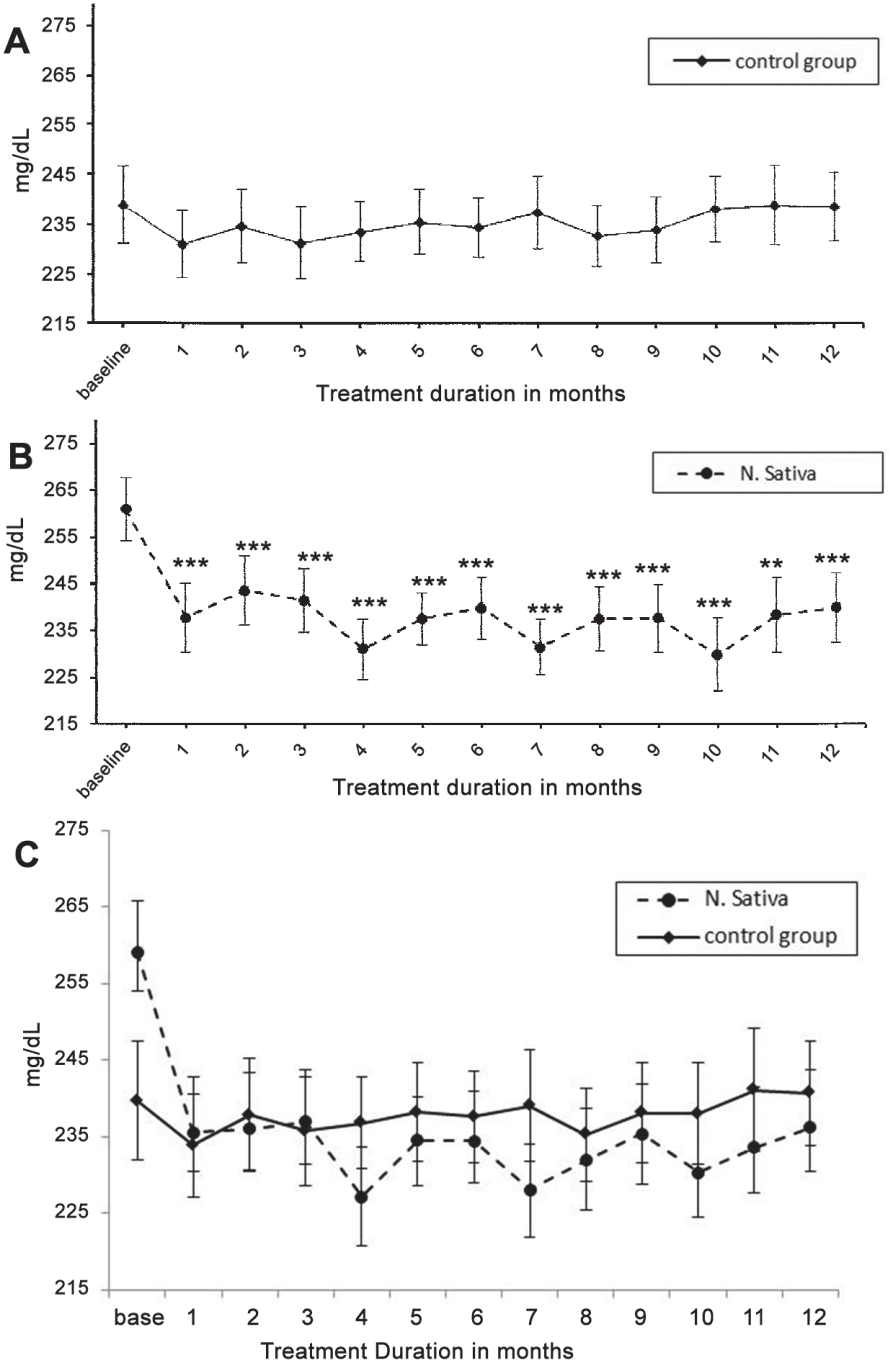
Changes in Random blood glucose (Mean±SEM) measured by self- monitoring glucometers, in the control (placebo) group and N. sativa group, compared to the corresponding baseline values using linear mixed model. Difference is significant at * P < 0.05, ** P <0.01 and *** P < 0.001.

Table 2 shows the changes in glycemic control parameters in both NS and control groups. In the control group HbA_1c_ and insulin resistance were increased significantly (p<0.05) at 6^th^ and 9^th^ months and C-peptide was significantly higher (p<0.04) at 9^th^ month, compared to their respective baseline values. However, these changes were not associated with significant changes in FBG and β-cell activity. In NS treated group, FBG, HbA_1c_ and insulin resistance were significantly lower, while β-cell activity was significantly (P<0.01) higher than the corresponding baseline values in all the four readings taken along the one year supplementation period. C-peptide level was decreased but did not approach the level of significance at all-time points. Glucometer readings revealed the same pattern of highly significant decrease in both FBG and RBG in NS group (p<0.001) and no significant change in control group compared to the baseline values (Fig. 2 & Fig. 3).

**Table 2:** Changes in Fasting blood glucose (FBG), Hemoglobin A_1c_ (HbA_1c_), C- peptide (C-pept), Insulin resistance (IR) and β-cell activity at different treatment durations, compared to their corresponding baseline values, in the control (placebo) and N. sativa groups.

	Duration of treatment in months (m)									
	Control Group					N. sativa group				
Variables	B (n = 52)	3 m (n = 52)	6 m (n = 51)	9 m (n = 48)	12m (n = 48)	B (n = 51)	3 m (n = 51)	6 m (n = 50)	9 m (n = 50)	12m (n = 48)
**FBG (mg/dL) Mean ± SEM**	180 ± 5.75	184 ± 5.81	185 ± 5.59	183 ± 5.41	180 ± 5.59	195 ± 6.57	163 ± 6.31	164 ± 5.97	176 ± 6.59	172 ± 5.83
**P**	—-	0.12	0.06	0.33	0.51	—-	0.002*	0.000*	0.021*	0.017*
**HbA**_**1c**_ **(%) Mean ± SEM**	8.2 ± 0.12	8.3 ± 0.12	8.3 ± 0.13	8.5 ± 0.15	8.5 ± 0.14	8.6 ± 0.13	7.9 ± 0.18	7.8 ± 0.22	7.9 ± 0.19	8.2 ± 0.14
**P**	—-	0.42	0.07	0.01*	0.06	—-	0.000*	0.000*	0.022*	0.010*
**C-Pept (ng/ml) (Mean ± SEM)**	2.9 ± 0.20	2.9 ± 0.19	3.0 ± 0.22	3.1 ± 0.19	2.8 ± 0.17	2.9 ± 0.20	2.9 ± 0.18	2.7 ± 0.17	2.7 ± 0.19	2.8 ± 0.17
**P**	—-	0.17	0.34	0.42	0.78	—-	0.63	0.21	0.13	0.12
**IR (Mean ± SEM)**	2.5 ± 0.17	2.6 ± 0.16	2.7 ± 0.19	2.7 ± 0.16	2.5 ± 0.15	3.0 ± 0.24	2.5 ± 0.16	2.4 ± 0.17	2.5 ± 0.19	2.5 ± 0.18
**P**	—-	0.07	0.04*	0.000*	0.26	—-	0.021*	0.022*	0.002*	0.004*
**β-cell (%) Mean ± SEM**	59.4 ± 4.93	57.8 ± 4.10	58.3 ± 4.34	59.7 ± 4.15	56.6 ± 3.51	45.8 ± 3.73	58.7 ± 5.17	57.6 ± 4.77	54.9 ± 3.39	58.6 ± 4.61
**P**	—-	0.35	0.54	0.75	0.54	—-	0.000*	0.001*	0.010*	0.011*

n = number of patients B = baseline values m = months

Analysis was carried out using Linear Mixed Model in SPSS.

*Difference is significant at P ≤ 0.05.

Comparison of the two groups for the five glycemic control parameters measured (Table 3) revealed a significantly lower HbA_1c_ in NS group than the control group at the 6 and 9 months readings. FBG followed the same pattern but was significantly lower at the 3 and 6 months readings. However the difference of C-peptide, insulin resistance and β-cell activity between the two groups was nonsignificant throughout the study period.

**Table 3:** Comparison of glycemic variables; fasting blood glucose (FBG), hemoglobinA_1c_ (HbA_1c_), C- peptide, insulin resistance, and β-cell activity between the control (placebo) group and N. sativa group at different treatment durations.

Variable (Means ± SEM)	Treatment duration	Control Group	N. sativa group	P value
**FBG (mg/dL)**	Baseline	180 ± 5.75 (52)	195 ± 6.57 (51)	0.06
	3 months	184.90 ± 5.81 (52)	163.82 ± 6.31(51)	0.02*
	6 months	185.80 ± 5.59 (51)	164.28 ± 5.97 (50)	0.01*
	9 months	183.88 ± 5.41 (48)	175.74 ± 6.59 (50)	0.34
	12 months	180.25 ± 5.59 (48)	172.52 ± 5.83 (48)	0.34
**HbA**_**1c**_ **(%)**	Baseline	8.2 ± 0.12 (52)	8.6 ± 0.13 (51)	0.44
	3 months	8.27 ± 0.12 (52)	7.89 ± 0.18 (51)	0.09
	6 months	8.34 ± 0.13 (51)	7.76 ± 0.22 (50)	0.03*
	9 months	8.47 ± 0.15 (48)	7.94 ± 0.19 (50)	0.03*
	12 months	8.48 ± 0.14 (48)	8.20 ± 0.14 (48)	0.17
**C- peptide (ng/ml)**	Baseline	2.9 ± 0.20 (52)	2.9 ± 0.20 (51)	0.92
	3 months	2.93 ± 0.19 (52)	2.85 ± 0.18 (51)	0.74
	6 months	3.02 ± 0.22 (51)	2.69 ± 0.17 (50)	0.25
	9 months	3.06 ± 0.19 (48)	2.72 ± 0.19 (50)	0.23
	12 months	2.84 ± 0.17 (48)	2.77 ± 0.17 (48)	0.76
**Insulin resistance**	Baseline	2.5 ± 0.17 (52)	3.0 ± 0.24 (51)	0.03*
	3 months	2.60 ± 0.16 (52)	2.52 ± 0.16 (51)	0.72
	6 months	2.69 ± 0.19 (51)	2.42 ± 0.17 (50)	0.27
	9 months	2.73 ± 0.16 (48)	2.48 ± 0.19 (50)	0.34
	12 months	2.51 ± 0.15 (48)	2.50 ± 0.18 (48)	0.95
β- **cell activity (%)**	Baseline	59.4 ± 4.93 (52)	45.8 ± 3.73 (51)	0.01*
	3 months	57.77 ± 4.09 (52)	58.65 ± 5.17 (51)	0.89
	6 months	58.34 ± 4.34 (51)	57.63 ± 4.77 (50)	0.91
	9 months	59.69 ± 4.15 (48)	54.86 ± 3.39 (50)	0.37
	12 months	56.62 ± 3.51 (48)	58.57 ± 4.61 (48)	0.74

Number of patients in parenthesis ()

*Differences are significant at P ≤ 0.05

Table 4 illustrates the changes in oxidant and antioxidant status in the control and NS groups. All readings in the control group were not changed significantly except at the 6 month where a significant (p<0.05) reduction in CAT and glutathione and elevation in TBARS was encountered. On the other hand, NS supplementation (2g/day) for one year induced a significant positive change in most readings taken for redox parameters. The first two readings (3 and 6 months) showed a significant increase in TAC (p<0.003), CAT (p<0.02), and a significant decrease in TBARS (p≤0.05). This positive effect was more pronounced in the subsequent two readings (9 and 12 months) where all parameters were significantly changed in the form of a significant elevation in TAC (p<0.002), SOD (p<0.04), CAT (p<0.003), and glutathione (p<0.03) and a significant reduction in TBARS (p<0.02).

**Table 4:** Changes in Total antioxidants capacity (TAC), Superoxide dismutase (SOD) activity, Catalase (CAT) activity, Glutathione and Thiobarbituric acid reactive substances (TBARS) at different treatment durations, in control (placebo) group and N. sativa group, compared to their corresponding baseline values.

	Duration of treatment in months (m)									
	Control Group					N. sativa group				
	B	3 m	6 m	9 m	12m	B	3 m	6 m	9 m	12m
Variables	n = 52	n = 52	n = 51	n = 48	n = 48	n = 51	n = 51	n = 50	n = 50	n = 48
**TAC (mM) Mean ± SEM**	2.5 ± 0.15	2.7 ± 0.13	2.5 ± 0.16	2.2 ± 0.13	2.3 ± 0.19	2.1 ± 0.17	2.8 ± 0.16	2.8 ± 0.14	2.8 ± 0.18	2.9 ± 0.12
**P**	---	0.20	0.76	0.12	0.77	---	0.000*	0.000*	0.000*	0.000*
**SOD (U/ml) Mean ± SEM**	2.3 ± 0.24	2.4 ± 0.25	2.4 ± 0.26	2.3 ± 0.26	2.3 ± 0.28	1.7 ± 0.18	1.8 ± 0.17	1.9 ± 0.22	1.9 ± 0.18	2.0 ± 0.19
**P**	---	0.32	0.43	0.42	0.34	---	0.43	0.049*	0.046*	0.023*
**CAT (nmol/min/ml) Mean ± SEM**	66.6 ± 4.98	68.6 ± 6.61	59.0 ± 5.17	63.0 ± 6.87	65.3 ± 5.18	55.0 ± 4.39	66.6 ± 4.25	68.1± 3.88	67.5 ± 4.40	71.7 ± 4.64
**P**	---	0.57	0.02*	0.19	0.24	---	0.011*	0.013*	0.000*	0.000*
**Glutathione (μM)Mean ± SEM**	3.3 ± 0.22	3.2 ± 0.24	2.7 ± 0.29	3.0 ± 0.20	3.0 ± 0.21	3.6 ± 0.26	3.8 ± 0.26	3.7 ± 0.25	4.4 ± 0.28	4.3 ± 0.23
**P**	---	0.55	0.02*	0.30	0.31	---	0.351	0.652	0.012*	0.006*
**TBARS (μM) Mean ± SEM**	48.3 ± 6.89	53.7 ± 5.77	54.2 ± 5.95	48.4 ± 5.86	52.9 ± 5.82	54.1 ± 4.64	44.6± 4.52	37.0 ± 3.04	41.2 ± 3.33	41.9 ± 3.16
**P**	---	0.08	0.05*	0.36	0.09	---	0.043*	0.001*	0.016*	0.013*

n = number of patients B = baseline values m = months

Analysis was carried out using Linear Mixed Model in SPSS.

*Difference is significant at P ≤ 0.05.

Comparison of the redox parameters between the two groups (Table 5) showed a significantly higher glutathione in NS group in all the 4 readings. Total antioxidant capacity was also higher in two readings (9 and 12 months). TBARS was significantly lower in NS group compared to control group in 6 and 12 months readings. The rest of readings were not significantly different comparing the two groups.

**Table 5:** Comparison of Total antioxidant (TAC) capacity, antioxidant enzymes; super oxide dismutase activity (SOD), catalase (CAT) activity, glutathione level and lipid peroxidation index (thiobarbituric acid reactive substances TBARS) between the control (placebo) group and N. sativa group at different treatment durations.

Variable (Means ± SEM)	Treatment duration	Control Group	N. sativa group	P value
**TAC (mM)**	Baseline	2.5 ± 0.15 (52)	2.1 ± 0.17 (51)	0.32
	3 months	2.69 ± 0.13 (52)	2.81 ± 0.15 (51)	0.56
	6 months	2.47 ± 0.16 (51)	2.76 ± 0.14 (50)	0.17
	9 months	2.18 ± 0.13 (48)	2.79 ± 0.18 (50)	0.01*
	12 months	2.33 ± 0.19 (48)	2.93 ± 0.12 (48)	0.01*
**SOC activity (U/ml)**	Baseline	2.3 ± 0.24 (52)	1.7 ± 0.18 (51)	0.11
	3 months	2.27 ± 0.25 (52)	1.75 ± 0.17 (51)	0.09
	6 months	2.39 ± 0.26 (51)	1.86 ± 0.22 (50)	0.13
	9 months	2.27 ± 0.26 (48)	1.89 ± 0.18 (50)	0.24
	12 months	2.34 ± 0.28 (48)	1.98 ± 0.19 (48)	0.29
**CAT activity (nmol/min/ml)**	Baseline	66.6 ± 4.98 (52)	55.0 ± 4.39 (51)	0.07
	3 months	68.64 ± 6.61 (52)	66.57 ± 4.25 (51)	0.79
	6 months	58.99 ± 5.17 (51)	68.06 ± 3.88 (50)	0.17
	9 months	62.98 ± 6.87 (48)	67.46 ± 4.40 (50)	0.58
	12 months	65.33 ± 5.18 (48)	71.73 ± 4.64 (48)	0.36
**Glutathione (μM)**	Baseline	3.3 ± 0.22 (52)	3.6 ± 0.26 (51)	0.12
	3 months	3.21 ± 0.24 (52)	3.79 ± 0.26 (51)	0.01*
	6 months	2.67 ± 0.29 (51)	3.68 ± 0.25 (50)	0.01*
	9 months	2.97 ± 0.20 (48)	4.39 ± 0.28 (50)	0.000*
	12 months	3.01 ± 0.21 (48)	4.31 ± 0.23 (48)	0.000*
**TBARS (μM)**	Baseline	48.3 ± 6.89 (52)	54.1 ± 4.64 (51)	0.09
	3 months	53.69 ± 5.77 (52)	44.58 ± 4.52 (51)	0.22
	6 months	54.15 ± 5.95 (51)	36.99 ± 3.04 (50)	0.01*
	9 months	48.38 ± 5.86 (48)	41.16 ± 3.33 (50)	0.28
	12 months	52.90 ± 5.82 (48)	41.88 ± 3.16 (48)	0.01*

Number of patients is given in parenthesis ().

*Differences are significant at P ≤ 0.05

All patients tolerated Nigella sativa treatment and there was no record of any side effect. Furthermore, results of renal functions, liver functions and complete blood count remained to be normal in all readings taken along the one year treatment with NS.

## Discussion

The results of the present study revealed that NS enhanced the glycemic control, manifested by the significant reduction in both FBG and HbA1cin all readings taken in the one year study duration, compared to the corresponding baseline. This reflects persistence of NS short term improvement in glycemic control (three months) in type 2 diabetic patients reported from our laboratory before.[18]

The reduction in blood glucose levels, encountered herein, agree with the findings of previous studies conducted on streptozotocin induced diabetic rats treated with crude NS[20], or with NS oil.[21] Also, reduction in HbA_1c_ as well as glucose levels were demonstrated in streptozotocin-nicotinamide induced diabetic rats supplied with thymoquinone.[22] Moreover, significant decline in blood sugar was recorded when NS volatile oil, 2.5 ml twice daily was added to metformin in the treatment of type 2 diabetic patients or those with insulin resistance syndrome.[17]

The mechanism of the hypoglycemic effect of NS has been suggested previously to be due to pancreatic actions via enhancing insulin secretion and inducing β-cell proliferation and regeneration.[9,11] Another group of researchers have proposed extra-pancreatic actions as a mechanism of NS hypoglycemic effect through enhancing tissue sensitization to insulin, especially liver and muscles.[10,12] Benhaddou-Andaloussi et al[12] have reported that NS ethanol extract greatly improves systemic glucose homeostasis in diabetic ‘*Meriones shawi*’ by acting through increasing circulating insulin and enhancing the sensitivity of peripheral tissues to the hormone. They attributed this effect, in part, to an activation of the AMPK pathway in skeletal muscle and liver; and to an increased content of Glut4 in skeletal muscle. In the present study, the recorded hypoglycemic effect of NS seems to be due to dual effect of this plant on peripheral insulin resistance and β-cell function. This is supported by the significant reduction in insulin resistance, and the significant elevation in β-cell function induced by NS supplementation in our study.

Oxidative stress and reactive oxygen species (ROS) have been proposed to be involved in the development of insulin resistance, β-cell dysfunction and diabetic complications.[23–25] The burden of production of these free radicals is largely counteracted by the antioxidant defense system, which includes the enzymatic scavenger superoxide dismutase, catalase, and glutathione peroxidase.[26] In the present study, NS supplementation to patients with type 2 DM for one year resulted in improved antioxidant status and reduction in oxidative stress, evident by significant increase in TAC and CAT activity associated with significant reduction in lipid peroxidation markers. This is the first report of such promising effect of NS in diabetic patients.

Our results are consistent with previous studies, conducted on streptozotocin-induced diabetic rats that reported lowered lipid peroxidation products and elevated levels of antioxidant enzymes; superoxide dismutase, catalase, and glutathione peroxidase in liver and kidney, after feeding with NS ethanol extract [15] and in pancreatic tissue after injection of NS oil.[14] Also, the potential role of thymoquinone, the active constituent of NS,in attenuating oxidative stress has been highlighted in streptozotocin-induced diabetic rats [27], and in hypercholesterolemic rats.[28]

Moreover, treatment with NS has been reported to improve the antioxidant status as well as antioxidant enzymes and decreased lipid peroxidation in humans exposed to pesticides-induced oxidative stress and hepatic toxicity.[29] However, Pourghassem-Gargari et al [30] found lower levels of lipid peroxidation but no differences in the levels of antioxidant enzymes; superoxide dismutase, and glutathione peroxidase, or in total antioxidant capacity, following oral feeding with crushed NS for one month in the hyperlipidemic rabbits. Based on the aforementioned data, we postulate that the hypoglycemic potential of NS may be attributed, at least partly, to reduced ROS with subsequent elevation of antioxidant defense system and suppression of lipid peroxidation; thus, preserving pancreatic β-cell integrity and increasing peripheral insulin sensitivity in patients with type 2 DM.

On the other hand, it has been reported that hyperglycemia in newly diagnosed type 2 diabetics caused the predominance of ROS over antioxidants, leading to oxidative DNA damage, which possibly contributed to pancreatic beta-cell dysfunction, insulin resistance and more pronounced hyperglycemia.[31] This vicious cycle finally induces the deterioration to diabetes. In view of the pivotal role of hyperglycemia in generation of excessive ROS and the subsequent oxidative stress in DM [2], and considering the hypoglycemic and antioxidant properties of NS recorded in this study, we postulate that NS, in addition to scavenging ROS, might also, improve the antioxidant status indirectly through lowering blood glucose level. In support, reduction in antioxidant enzyme activity has been reported to be related to poor glycemic control in diabetics.[32] Recently, Fiorentino et al[1] postulated that appropriate glycemic control, in which both hypoglycemic and hyperglycemic episodes are reduced, in association to the treatment of dyslipidemia, and hypertension, can counteract oxidative stress and, therefore, both microvascular and macrovascular complications of diabetes mellitus.

### Limitations

The major limitation to our study is this that we could not maintain diet and exercise records of the patients due to the length of study. Both of these factors can have confounding effect on the results. However as the patients came were asked to come fasting on the follow up, therefore the effect of diet will probably be minimal. Likewise although we tried our level best for standardization of self-testing of blood glucose by providing glucometers and calibrations, yet we cannot be sure about standardization. In addition the baseline values for Insulin resistance (IR) and β-cell function (that were calculated using computer software HOMA2, and shown in table 2) were significantly different between the two groups. However we could not single out any outliers that led to this difference. This factor must have affected the significant improvement shown for the NS group.

## Conclusion

Nigella sativa improves glycemic control and ameliorates oxidative stress in patients with type 2 diabetes mellitus. The present study supports the results of our previous studies, indicating the favorable effect of NS on glucose homeostasis that persisted along the one year study period. Therefore, NS has the potential to be used as a natural adjuvant to oral glucose lowering drugs in the management of type 2 diabetes mellitus. As NS is a very low cost herb, therefore the potential cost benefit ratio will be in favor of benefit. Further phase III clinical trials are recommended to move forward in this promising area of research.

## Supporting Information

S1 TREND Checklist (PDF)
